# The Ca^2+^-sensing receptor and the pocketome: comparing nature’s complexity with human intervention in receptor modulation

**DOI:** 10.1038/s41392-024-01863-8

**Published:** 2024-07-15

**Authors:** Edda S. F. Matthees, Carsten Hoffmann

**Affiliations:** 1 Institut für Molekulare Zellbiologie, CMB—Center for Molecular Biomedicine, Universitätsklinikum Jena, Hans-Knöll Straße 2, D-07745 Jena, Germany; 2 Friedrich-Schiller-Universität Jena, Hans-Knöll Straße 2, D-07745 Jena, Germany

**Keywords:** Structural biology, Biochemistry

In a recent paper published in Nature,^1^ the relatively understudied Ca^2+^-sensing receptor (CaSR) within the G protein-coupled receptor (GPCR) family C, gained attention due to pioneering research led by Georgios Skiniotis from Stanford University, with four shared first authors. Their international team unveiled cryogenic electron microscopy (cryo-EM) structures of the human CaSR, embedded in lipid nanodiscs, elucidating its interaction with the calcimimetic drug cinacalcet, G_i_ or G_q_ proteins and provided structural insights into gain or loss of function mutations associated with human diseases.^1^

Similar to other family C receptors such as metabotropic glutamate (mGlu) or γ-aminobutyric acid B (GABA_B_) receptors, the CaSR features a large N-terminal extracellular ligand-binding domain alongside the classical seven transmembrane (7TM) helical domain. However, until now, its activation mechanism remained unknown, evoking questions about its similarity to other class C GPCRs.^2^ In their recent publication, He et al. reveal that the CaSR forms an asymmetrically activated dimer, mirroring the activation pattern observed for other class C family members.^1,2^ In this dimer, G proteins selectively engage one of the two protomers, inducing significant rearrangements within the intracellular loop (ICL) 2, in contrast to the major rearrangements observed in TM6 and ICL3 commonly seen in many class A family GPCRs. This structural insight clarifies why FRET-based conformational change sensors for family C GPCRs specifically require fluorophore insertion in ICL2 rather than ICL3.^3^ Nevertheless, receptor activation is not solely induced by agonist binding, but requires G protein interaction to achieve full activation, a mechanistic feature shared across different GPCR families, as exemplary observed for class A receptors. Here, the authors clearly elucidate that the coupling selectivity towards G_i_ or G_q_ proteins is determined by differential structural rearrangements in the ICL2 along with the receptor C-terminus, collectively forming the respective binding interface for the G protein.^1^ Additionally, the binding of a G protein induces a distinct asymmetric rearrangement of the dimer interface compared to the inactive receptor dimer. Recent findings suggest that such dimer rearrangements are temporally positioned between the steps of G protein binding and activation.^4^ It is tempting to speculate that such kinetic orders might be a common feature among dimeric GPCRs.

**Fig. 1:**
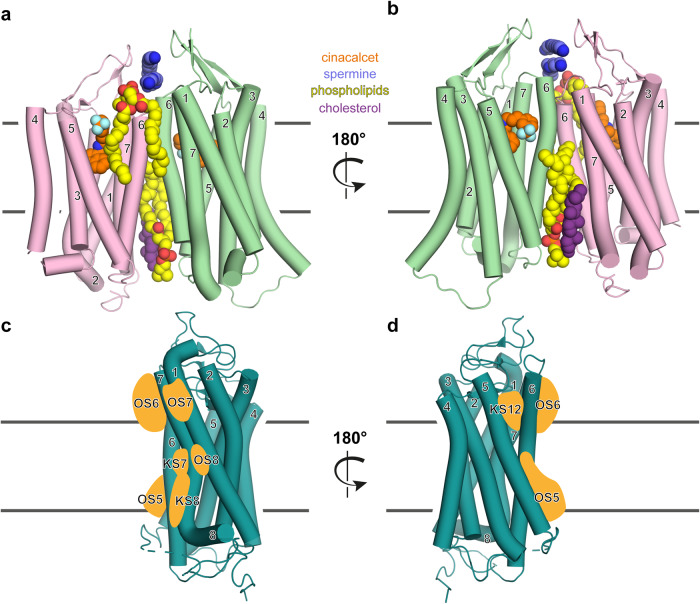
Exploring allosteric CaSR ligand binding and the GPCR pocketome. **a**, **b** Cryo-EM structure of the CaSR dimer with numbered TM helices, similarly color-coded as in the original study.^1^ The active protomer, interacting with the G protein (not shown) is illustrated in green and the inactive protomer in pink. The large extracellular ligand binding domain is not shown to enable a detailed depiction of the classical 7TM helical domain, similar to d, e in the original publication.^1^ In the structure many different interacting molecules were resolved: The calcimimetic drug cinacalcet (orange, blue), located in a comparable orthosteric binding pocket of other GPCR families, spermine (dark blue), above the TM dimer interface, phospholipids (yellow, red) at the front of the dimer (**a**), as well as the back of the dimer (**b**), in addition to cholesterol (purple). PDB: 8SZG, 8SZH. X-ray structure of a class A GPCR with numbered TM helices from front (**c**) and back (**d**), similarly oriented as the active protomer (green) above (**a**, **b**). Based on the analysis of more than 500 class A GPCRs and their pocketome of allosteric ligand binding sites by ref. ^5^ potentially analogous utilized binding sites in the class C CaSR structure are schematically represented as orange shapes (orphan site (OS) 5 (middle and lower portion (MP, LP, respectively) TMV, TMVI), OS6 (upper portion (UP) TMVI, TMVII), OS7 (UP TMI, TMVII), OS8 (MP TMI, TMVII); known sites (KS) 7 (MP TMVI, TMVII), KS8 (LP TMVI, TMVII), KS12(UP TMV, TMVI))

In the complex structures of the CaSR with G_i_ or G_q_ protein, multiple binding sites for different ligands are resolved, ranging from the orthosteric ligand-binding Venus flytrap domain to allosteric modulators binding to the classical 7TM domain.^1^ To enable a detailed localization of the utilized allosteric ligand interaction interfaces, only the 7TM domain of the dimer is depicted in Fig. 1a, b. Both the active (green) and inactive (pink) protomers bind cinacalcet (orange, blue). However, the authors observed distinct structural characteristics of the bound cinacalcet, with it appearing extended in the inactive protomer and bent in the active one. The modulatory ligands like spermine (dark blue), phospholipids (yellow, red) or cholesterol (purple), specifically, bind to distinct positions at the extracellular part of the 7TM helical domain or within the dimer interface, seemingly greasing the gears of dimer interface rearrangements and potentially stabilizing the ligand-specific dimer conformations.

Upon comparing the approximate interaction sites of these modulatory ligands, they correspond well with several predicted allosteric sites on GPCRs (Fig. 1c, d), based on an extensive analysis of over 500 class A GPCRs, proposing the GPCR ‘pocketome’ as the ensemble of all potential binding sites on GPCRs.^5^ This observation gratifyingly suggests that promising pharmacologically untargeted (orphan) sites, as identified by ref. ^5^ are naturally utilized to fine-tune receptor activity across families. Furthermore, He et al. underscore this in a pathophysiological context by examining the role of amino acids within the spermine-binding site. As a loss of function example, individuals with autosomal dominant hypocalcaemia type 1 (ADH1) were shown to have mutations in the spermine binding site, resulting in the substitution of the negatively charged glutamic acid to the positively charged lysine, which presumably disrupts binding of polyamine spermine.^1^

Overall, the publication by He et al. holds great interest for the research community, as it not only confirms and extends various characteristics observed in other members of the GPCR family C but also indicates mechanistic features shared across different GPCR families. Furthermore, their study elegantly demonstrates how structural information can help to elucidate disease mechanisms.

### Supplementary information

Author Checklist
